# Prenatal Alcohol Exposure and the Development of Multiple Risk Behaviors in Adolescence: A Birth Cohort Study

**DOI:** 10.1111/acer.70286

**Published:** 2026-05-01

**Authors:** James T. I. Parsonage, Laura Tinner, David Troy, Caroline M. Taylor, Cheryl McQuire

**Affiliations:** 1 School for Public Health Research (SPHR), National Institute of Health and Care Research (NIHR), Newcastle, UK; 2 Centre for Public Health, Bristol Medical School, University of Bristol, Bristol, UK; 3 Population Health Sciences, Bristol Medical School, University of Bristol, Bristol, UK; 4 Applied Research Collaboration West, National Institute of Health and Care Research ( NIHR ARC West), Bristol, UK; 5 Centre for Academic Child Health, Bristol Medical School, University of Bristol, Bristol, UK

## Abstract

**Background:**

The United Kingdom has the fourth highest estimated prevalence globally of maternal alcohol consumption during pregnancy (41%). It is therefore important to understand the long-term impacts of prenatal alcohol exposure (PAE) by examining its impact on the development of adolescent multiple risk behaviors (MRBs) that may increase morbidity and premature mortality across the life-course.

**Methods:**

Using Avon Longitudinal Study of Parents and Children (ALSPAC) cohort data with multiple imputation (*n* = 6752), we examined the impacts of “infrequent,” “frequent,” and “binge” PAE groups on the development of seven MRBs at 16 years old, encompassing substance misuse, risky sexual behavior, and antisocial behavior. Data were analyzed using multiple regression, using q-statistics to adjust for multiple comparisons.

**Results:**

Adolescents with “infrequent” and “frequent” PAE were more likely to develop hazardous alcohol use at 16 years old compared to those without PAE, with the strongest association being for the “frequent” group (adjusted odds ratio [aOR] 1.45 [1.19–1.76], *p* < 0.001, *q* value = 0.005). Adolescents exposed to binge drinking prenatally had an increased risk of engaging in underage sexual intercourse (aOR 1.34 [1.09–1.64], *p* = 0.005, *q* value = 0.044). Binge drinking predicted a higher total MRB score (Coefficient = +0.21 [+0.08 to +0.33], *p* = 0.001, *q* value = 0.017).

**Conclusions:**

This study supports the UK Chief Medical Officer’s Low Risk Drinking Guidelines that the safest approach if pregnant, or if there is a possibility of becoming pregnant, is to avoid drinking alcohol, with the more alcohol consumed during pregnancy the greater the risks of long-term harm to the baby. Given the findings that PAE may increase the risk of adolescent hazardous alcohol use and risky sexual behavior, this study highlights the need for further research to understand the intergenerational effects of PAE.

## 1 Introduction

The United Kingdom Chief Medical Officer’s (CMO) guidelines advise that the safest approach to mitigate the short and long-term risks of alcohol consumption during pregnancy is to avoid alcohol altogether (Public Health England 2020). The prevalence of alcohol consumption during pregnancy in the United Kingdom has been estimated to be about 41%, one of the highest estimated percentages in Europe, which has a regional prevalence of fetal alcohol spectrum disorders (FASD) about 2.6 times higher than the global average (Popova et al. 2017). The prevalence of FASD has been estimated in UK school children at 1.8% and, when including “probable” cases, was 3.6% (McCarthy et al. 2021).

Prenatal alcohol exposure (PAE) is a key factor in the diagnostic criteria for FASD and those “at risk of neurodevelopmental disorder or FASD” (Scottish Intercollegiate Guidelines Network [hereafter SIGN] 2019). The neurodevelopmental disorder of FASD results in lifelong impairment and may present as problems with attention and cognition, social impairment, and difficulties regulating behavior. For those with FASD, adolescence represents a crucial period where neurodevelopmental impairments meet growing societal pressures in the transition to adulthood, increasing the need for high intensity support (McLachlan et al. 2020). Exposure to and uptake of risk-taking behaviors may begin in adolescence as individuals develop greater autonomy over life choices affecting their health (Campbell et al. 2020).

The long-term impact of PAE on children in adolescence and beyond may be examined by using multiple risk behaviors (MRBs) as outcomes. MRBs develop or increase throughout adolescence and can prevail throughout life, leading to future morbidity and premature mortality (Wright et al. 2018). The risk to an individual’s health accumulates based on the number of distinct MRBs performed rather than the pattern of different behaviors shown (Wright et al. 2020). As such, MRBs can be considered both as a total number of distinct risk behaviors, as well as individual behaviors that meet a threshold considered hazardous to an individual’s health.

The etiology and occurrence of risk behaviors has been well explored in the literature on FASD. Individuals with FASD may struggle with executive functioning resulting in difficulties with socialization, impulse control, and cause and effect thinking, and some may express behaviors, including aggressiveness, criminal activity, and sexual inappropriateness (Rasmussen et al. 2008). A recent meta-analysis found that conduct disorders, alcohol and drug dependence, and disturbances of activity or attention were extremely common in those with FASD (Popova et al. 2016). The Raine study found that four or more Australian standard drinks of alcohol per week (100 mL of wine or equivalent) during pregnancy led to an increased risk of adolescent harmful alcohol use, compared with no PAE (Duko et al. 2020). The Mater University Study of Pregnancy found that three or more glasses a few times per month during pregnancy were correlated with alcohol use disorders in adolescence (Alati et al. 2006). Individuals with FASD also experience many secondary difficulties throughout their lives, such as meeting independent living needs (Olson and Sparrow 2021). It is essential that strengths-based approaches and interventions are developed to improve the quality of life of those with the condition and the difficulties associated with it. This includes removing system-level barriers experienced by many families, carers, and individuals with FASD (Petrenko et al. 2014). However, the need to understand the impacts of PAE remains, to target and improve services and approaches that support the health of those with FASD or PAE.

This study addresses this need by bringing the adolescent impacts of PAE into sharper focus and is the first to quantify the impacts of PAE with respect to a broad range of MRBs. Using Avon Longitudinal Study of Parents and Children (ALSPAC) birth cohort data, this observational study aimed to examine seven MRB outcomes for adolescents with PAE. Our primary aim was to examine the impact of different patterns of PAE, including binge drinking, on the total number of MRBs, encompassing substance use, antisocial behavior, and risky sexual behavior, displayed by age 16 years. Our secondary aim was to look at how different patterns of PAE impact the development of each individual risk behavior.

## 2 Materials and Methods

### 2.1 Overview of the Data Source and Study Design

Pregnant women resident in Avon, UK, with expected dates of delivery between April 1, 1991, and December 31, 1992, were invited to take part in the study with the initial number of pregnancies enrolled being 14,541. However, the total sample size for analyses using any data collected after the age of seven is 15,447 pregnancies (15,658 fetuses) due to subsequent recruitment (Boyd et al. 2013; Fraser et al. 2013; Northstone et al. 2023). Please note that the study website contains details of all the data that are available through a fully searchable data dictionary and variable search tool (University of Bristol 2025).

**Figure 1:**
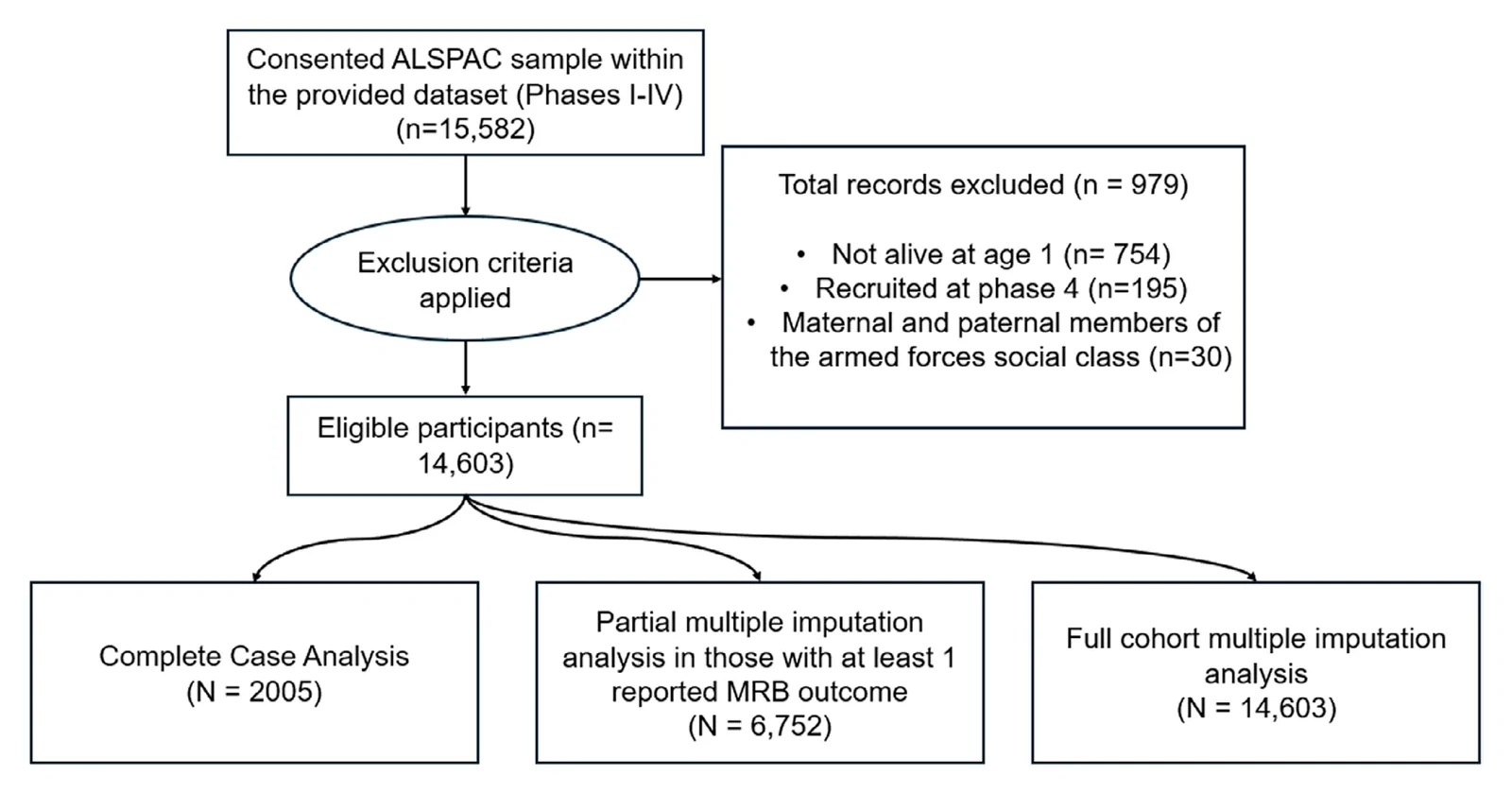
Participant flow diagram.

From an initial sample of 15,582 consented birth records, we excluded records of: (a) participants from Phase IV (children enrolled after the age of 18), (b) children who died before 1 year of age, and (c) mothers and partners assigned to the “armed forces” social class, due to sparse data. Fourteen thousand six hundred and three linked records of mothers, partners, and their children remained eligible as shown in Figure 1. Maternal, paternal, and child data were gathered via questionnaires and clinic assessments.

**Figure 2:**
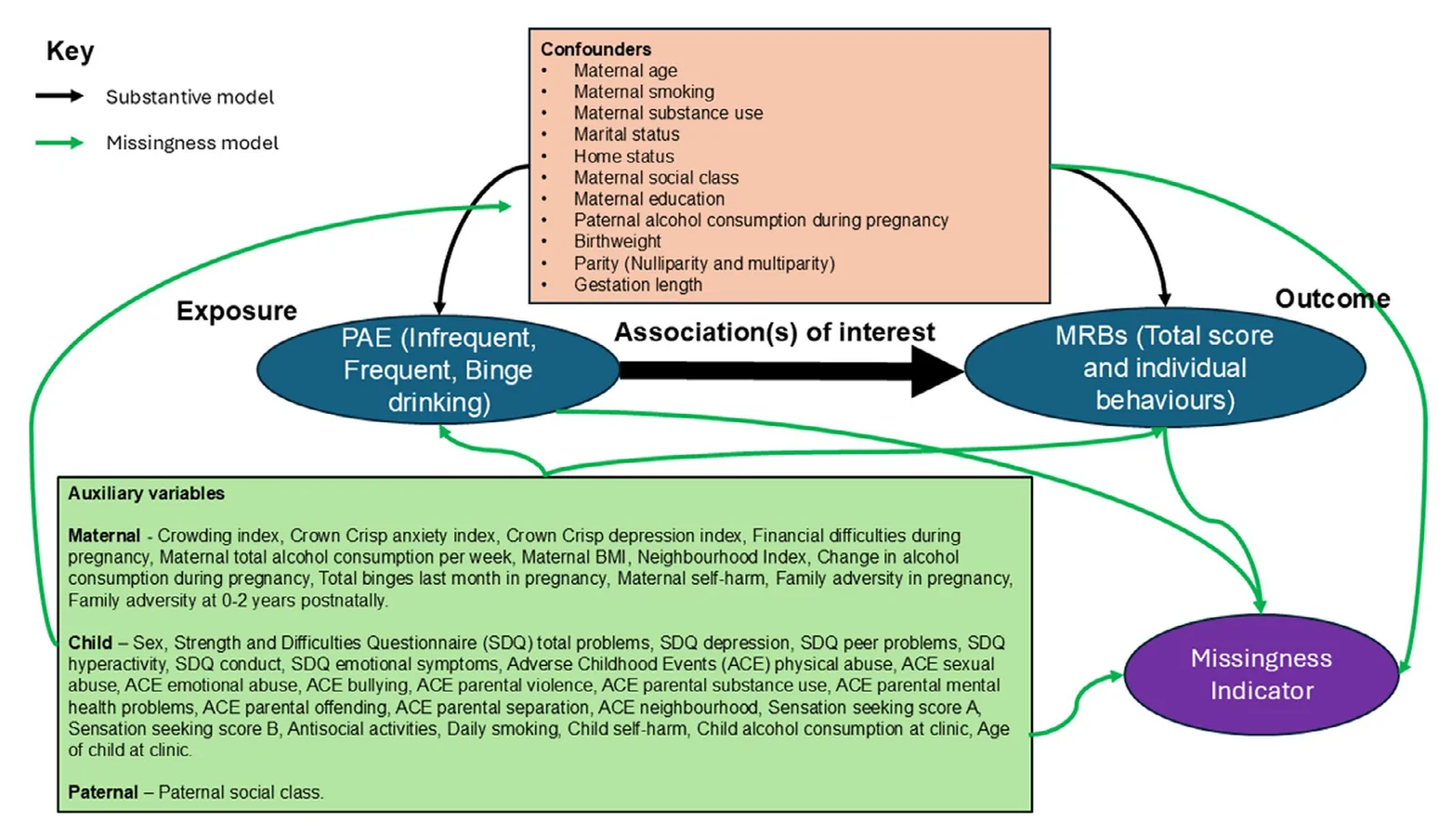
Included substantive model and missingness model variables.

We first analyzed data using a complete-case strategy, where only those with available data for all exposure, confounder, and outcome variables were included. This resulted in a considerably attenuated sample size of 2005 participants. To increase precision, explain missingness, and address the notable bias in prenatal binge-drinking exposure, maternal and paternal covariates and MRB outcomes when conditioning on being a complete case (shown in Table S2), we performed multiple imputation under the assumption that the data were missing at random. We performed two imputation strategies, a partial imputation where individuals had at least one complete MRB outcome (*n* = 6752) and a full imputation for all eligible records in the ALSPAC cohort (*n* = 14,603). There was substantial missingness found in the outcome MRBs, ranging between 64.7% and 69.0% of records in the full imputation. Given concerns around the degree of outcome missingness and therefore amount of imputed data in the full imputation, the partial multiple imputation analysis is considered the primary analysis. Results for the complete-case analysis are provided in Tables S3 and S4. The full ALSPAC imputation results are shown in Tables S7 and S8. Figure 2 shows the substantive model and missingness model variables which are part of the complete-case strategy and subsequent multiple imputation analyses.

### 2.2 Ethics Statement

The ALSPAC Executive Committee approved the study proposal on April 29, 2024 (Proposal identifier: B4600). Ethics approval for the study was obtained from the ALSPAC Ethics and Law Committee and the Local Research Ethics Committees. Informed consent for the use of data collected via questionnaires and clinics was obtained from participants following the recommendations of the ALSPAC Ethics and Law Committee at the time.

### 2.3 Exposure Variables

Table 1 shows exposure definitions based on the timing, amount, and frequency of PAE, measured by a self-reported maternal questionnaire at 18-week gestation. Two exposure types were examined in this study. The first was increasing frequency of PAE grouped as follows: (i) No PAE reported, (ii) infrequent PAE (< 1 glass of wine per week or equivalent measure), and (iii) frequent PAE (≥ 1 glass of wine per week or equivalent measure). The second was prenatal binge-drinking exposure grouped as follows: (i) no binge drinking reported and (ii) binge drinking (≥ 2 pints of beer or equivalent measure on one or more occasion).

### 2.4 Outcome Variables

Outcomes were seven MRBs encompassing substance use, antisocial, and risky sexual behavior. Hazardous alcohol use, smoking, cannabis use, and drug use (excluding cannabis) outcomes were derived from a child self-reported questionnaire at age 16. Antisocial behavior, underage sexual intercourse, and unprotected sexual intercourse outcomes were derived from questions asked at clinic visits at age 15.5 years. Hazardous alcohol use questions collectively form the AUDIT score measure, a validated measure of problem drinking in adolescent populations (Rumpf et al. 2013). All other outcomes use standardized MRB definitions, described in prior scientific literature (Wright et al. 2020). The definitions of each outcome are described in Table 2.

### 2.5 Covariates and Confounding

**Figure 3:**
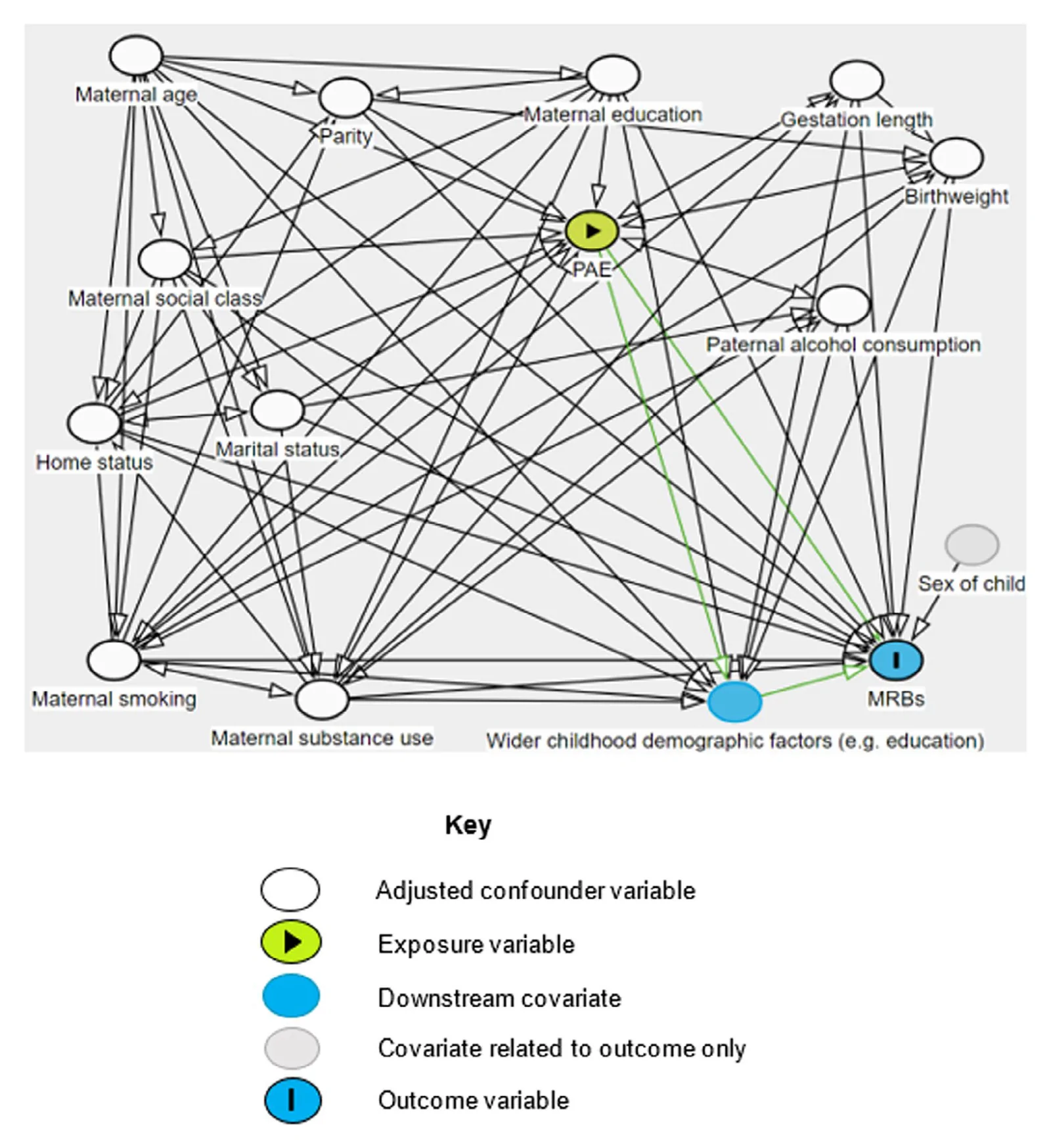
Directed acyclic graph of confounding covariates between PAE and MRBs.

Drawing on the literature, we developed a directed acyclic graph (DAG) using the DAGitty software to depict hypothesized causal and confounding pathways and inform covariate selection to minimize bias in our substantive models (Figure 3) (McQuire et al. 2019; Textor et al. 2016). Covariates used for model adjustment were maternal age, parity, birth-weight, maternal social class, marital status, home ownership status, highest maternal educational qualification, maternal smoking, maternal cannabis use, maternal drug use (excluding cannabis), and paternal alcohol use during pregnancy. Likelihood ratio tests, for each MRB, were used to ascertain that the adjusted model was a better fit for the observed data than an unadjusted model.

### 2.6 Descriptive Statistics

Tables 3 and 4 show pooled descriptive statistics from the multiple imputation. We calculated descriptive statistics for the complete cases using the “arsenal” R package (Heinzen 2016). Descriptive statistics for complete cases and non-complete cases are presented in Tables S1 and S2, respectively.

### 2.7 Multiple Imputation

Variables for multiple imputation models included hypothesized risk factors for PAE and MRBs, sociodemographic variables, and auxiliary variables (i.e., variables that are not included in the main analyses of interest, but which may improve the performance of missing data models) (see Figure 2) (Collins et al. 2001). Through systematically appraising the ALSPAC variable catalog, a wide range of variables were selected which were considered to help explain missingness in substantive model variables. The “quickpred-pt2” method was then used to assign variables to the imputation model with a correlation cut off (“mincor”) of 0.2. The partial imputation presented here included all records with at least one MRB outcome (*n* = 6752) and therefore removes all individuals who had no MRB data.

Multiple imputation was performed using the MICE package in the R software (R Core Team 2023; van Buuren and Groothuis-Oudshoorn 2011). For the MRB score analysis, 70 imputations were performed across 80 iterations for stability. For the individual MRB analysis, 50 imputations were performed across 50 iterations for stability. Rubins rules were applied to pool estimates across imputations for the multiple linear and logistic regression analyses. Further information about the imputation models, MICE methods, and diagnostics can be found in the Supporting Information.

### 2.8 Analysis

We analyzed the imputed data using multiple regression to quantify the strength of the associations between PAE and the following outcomes at age 16 years: (1) the adjusted mean difference in MRB score in the PAE exposure groups compared with comparator groups (ranging from 0 to 7 MRBs); (2) the relative likelihood (adjusted odds ratio [aOR]) of participants displaying each component MRB between PAE exposure and comparator groups. We applied *q* values to results to account for multiple comparisons (Storey 2011).

## 3 Results

### 3.1 Descriptive Statistics

Within the multiple imputation sample (Table 3), 66.4% of mothers reported at least infrequent PAE. Mothers of children with increasing frequency of PAE were more likely to be older, to smoke, use cannabis, be unmarried, have higher educational qualifications, to be part of social classes I to III (Non-manual), and to have partners who drank more frequently during the pregnancy. Multiparity was more prevalent in mothers of children with frequent PAE, whereas nulliparity was more prevalent in mothers of children without PAE. The strongest association to increasing maternal alcohol consumption was their partner’s alcohol consumption.

A similar direction in associations was seen in mothers of children with binge-drinking exposure compared with those without binge-drinking exposure, with regards to maternal smoking, substance use, and marital status. However, higher percentages of mothers of children with binge-drinking exposure were from social classes III-Manual to V (unskilled manual) and had a highest education qualification below A-level than mothers of children unexposed to binge drinking. There were no meaningful differences in maternal age at delivery and parity between mothers of children with binge-drinking PAE compared with those who were unexposed to binge drinking.

### 3.2 Multiple Regression

#### 3.2.1 Analysis 1: Adolescent MRB Scores and PAE

There was no strong statistical evidence that children with “infrequent” or “frequent” PAE exhibited more MRBs at age 16 years than those without PAE (Table 5).

There was statistical evidence of a positive association between exposure to prenatal binge drinking (defined as two or more pints of beer or pub measure equivalent on at least one occasion) and MRBs in adolescents. Adolescents who were exposed to binge drinking prenatally had a small increase in adjusted mean MRB score (+)0.21 (+0.08 to +0.33), *p* = 0.001, *q* = 0.017 compared with those unexposed to binge drinking.

#### 3.2 Analysis 2: Individual Risk Behavior Development at Adolescence and PAE

Table 6 shows the increased likelihood of hazardous alcohol use in the past year from age 16 years in adolescents with “frequent” PAE, defined as ≥ 1 glass of wine per week or equivalent pub measure (aOR 1.45, 95% CI 1.19–1.76, *p* < 0.001, *q* value = 0.005), compared with adolescents without PAE (Table 6). No evidence was found of an increased likelihood of developing other MRBs at age 16 in either the “infrequent” or “frequent” PAE groups compared with adolescents without PAE.

Adolescents exposed to binge drinking during pregnancy were more likely to engage in underage sexual intercourse by age 16 (aOR 1.34, 95% CI 1.09–1.64, *p* = 0.005, *q* = 0.044) than adolescents unexposed to binge drinking during pregnancy. No *q* value supported evidence was found that prenatal binge-drinking exposure increased the likelihood of developing other MRBs at age 16 years.

## 4 Discussion

This study introduces important risky sexual behavior outcomes, which were not included in a recent systematic review of ALSPAC studies investigating the link between PAE and childhood outcomes (Ogunjimi et al. 2025). It also provides different findings to the one study in the review investigating the association between PAE and problematic adolescent alcohol use which previously found that only in univariate analysis, maternal alcohol consumption was only related to adolescent alcohol consumption and adolescent alcohol problems (Kendler et al. 2013). However, differences may be explained by our use of a different outcome threshold, adolescent hazardous alcohol use (AUDIT score of 8 or more), and in our use of different ordinal groups by both amount, type and timing of PAE.

In our multivariate analysis, at a *q* value level, the strongest relationship found was between exposure to frequent PAE (≥ 1 glass of wine per week or equivalent measure) and having higher odds of reported hazardous alcohol use at age 16. Binge-level PAE was associated with a small increase in overall number of risk behaviors developed and underage sexual intercourse in adolescence. As well as the increase in mean adolescent MRB scores that we observed in the binge-drinking group in our study, the specific combination of types of MRB observed—hazardous alcohol use in the frequent drinking group and underage sexual intercourse in the group exposed to binge drinking prenatally—are of particular importance. Concurrent hazardous alcohol use and underage sexual intercourse in adolescence may perpetuate intergenerational transmission of PAE and alcohol misuse.

It is also important to note that the sample prevalence of PAE estimated using multiple imputation in this study (66.4%) is likely reasonable when considering a modern UK prevalence of 41% may be considered conservative, with the upper bounds of modern estimates being up to 75% (Popova et al. 2017).

The exposure and comparator group descriptive differences are not unexpected. Increasing alcohol consumption is widely linked with smoking and cannabis use. Greater maternal age has also been associated with increased alcohol consumption in pregnancy (Meschke et al. 2013). In a US survey, those who were not married, were employed, and had higher educational attainment were more likely to report alcohol consumption during pregnancy (Gosdin et al. 2022). Binge drinking during pregnancy has also been associated with lower educational attainment and lower paid employment (Currie et al. 2020).

The binge-drinking definition derived from ALSPAC questionnaires, namely “two pints of beer or more or equivalent on one occasion” (roughly between four and six units depending on the alcohol concentration), includes a lower unit range than the current definition of binge drinking for women (six units on one occasion) (Department of Health and Social Care [hereafter DHSC] 2021). Given the findings within this study, we may hypothesize that the observed associations between PAE and MRBs may be stronger at the current definition level as greater alcohol exposure would likely be more harmful to the developing fetus.

There are multiple biologically plausible and explanatory theories for associations between PAE and adolescent risk behavior development. Alcohol is a known teratogen, which can lead to abnormal brain development in utero (Chung et al. 2021). Maternal genotypes can also influence alcohol drinking behavior and alcohol metabolism (Sambo and Goldman 2023). These factors, coupled with certain infant genotypes thought to influence susceptibility to harmful PAE effects, may influence the likelihood of FASD being developed (Gemma et al. 2007). It is also hypothesized that epigenetic processes, such as alterations in DNA methylation and histone methylation, which influence gene expression, continue to influence brain development long after initial PAE (Jarmasz et al. 2019). Studies in rats, for example, have shown that PAE potentiates increased alcohol consumption in the offspring at adolescence (Fabio et al. 2015; Marengo et al. 2025).

### 4.1 Study Strengths and Limitations

The key strengths of this study include the use of a large population-based cohort with prospective, detailed measurement of PAE. This enabled investigation of different patterns, doses (including low doses), and frequency of alcohol exposure on a range of MRB outcomes.

Given the range of MRB outcomes examined, using a *q* value < 0.05 threshold to account for false discovery rates reduces the potential for Type 1 errors in our results. Associations detected at a *p* value < 0.05 but with attenuated *q* values (≥ 0.05)—such as associations between infrequent PAE and adolescent hazardous alcohol use—may still reflect true effects. However, because multiple hypotheses were tested, the risk of Type I error is elevated and, compared with *q* values, unadjusted *p* values provide less certainty that these findings are not due to chance.

We must note that, as questionnaire responses for PAE were based on responses in Trimesters 1 and 2 of pregnancy, we can draw no clear conclusions about the impacts of PAE before conception or during Trimester 3. In addition, despite the use of a DAG to model variable relationships in the substantive model, we must be aware that residual confounding may exist that may influence associations between PAE and MRBs.

As there is also the potential for ongoing maternal/paternal drinking and the clustering of these with other factors in the postnatal environment (e.g., other lifestyle factors), it is unclear whether the effects demonstrated in this study are directly due to the biological impacts of alcohol in utero or the effects of the postnatal environment. Future research may wish to use other causal inference techniques (e.g., paternal controls) to help elucidate the relative contribution of prenatal and postnatal factors on the outcomes we have observed in adolescents in this study. Inclusion of polygenic scores for alcohol use should also be considered to help address residual confounding. A further option is to use a within-family study design comparing outcomes in siblings who were exposed to varying levels of PAE.

Attrition within the ALSPAC cohort has been shown to be associated with low socioeconomic status (SES) leading to underestimation of the effects of inequalities (Howe et al. 2013). It is unclear what impacts this attrition would have had on our study, given that higher SES in our sample was more associated with regular, sustained PAE. However, given that low SES may be related to prenatal binge-drinking exposure and hazardous alcohol use in adolescence, this may have led to underestimation of the impacts of prenatal binge-drinking exposure on MRBs (Katikireddi et al. 2017). Given the neurobehavioral impacts of FASD, it could also be hypothesized that those most impacted by PAE may be those least likely to repeatedly respond to the questionnaires, leading to further attrition bias. This may be reflected in results through both a reduction in the strength and size of the association between PAE and MRB development. The use of multiple imputation in this study will have helped mitigate the impacts of selection bias due to missing data and will have improved precision in our estimates.

Underestimation of alcohol consumption is a known issue when using self-report measures. This has been demonstrated in studies that compare reported alcohol use in questionnaires with sales data (Stockwell et al. 2018). Considerable stigma experienced around the impacts of PAE may further impact information disclosure about both alcohol and wider substance use (Stone 2015). The low thresholds set here for inclusion in the “infrequent” PAE group (less than one glass of wine or equivalent per week) are both a strength and limitation, by capturing more of those underestimating their intake but also potentially leading to an overestimation of the impacts of lower reported levels of PAE.

When defining our exposure groups, we found that stratifying the “frequent” PAE group into more categories was not deemed possible due to the rarity of higher levels of reported PAE leading to inadequate sample sizes for analysis. Although potential effects of unit increases may have been explored, very few mothers reported drinking more than two units per week. As such, focusing on a stratified unit score may have resulted in inadequately sized groups for analysis. There was also substantially more missingness in the continuous measure for PAE than the selected PAE variables.

Finally, the use of a continuous outcome variable (the MRB score) to summarize occurrences of distinct risk behaviors is useful in examining the overall number of risk behavior differences between exposure groups. However, the overall score, particularly after model adjustment, obfuscates the individual MRB components contributing to it. This continuous score also implicitly assumes that all MRBs can be considered equivalent. This represents an oversimplification in terms of health impacts. We addressed this by examining the likelihood of individual MRBs occurring in those with different PAE profiles.

### 4.2 Implications

This study supports current UK Chief Medical Officers (CMOs) guidance that the safest approach if pregnant, or planning a pregnancy, is to not drink alcohol. Adolescents in our study had an increased risk of engaging in harmful alcohol use when exposed prenatally to the equivalent of one or more drinks of alcohol per week. In addition, adolescents exposed prenatally to binge-level alcohol exposure (here defined as two pints of beer or equivalent on one or more occasion) were at increased risk of engaging in underage sexual intercourse and had higher MRB scores than those who were unexposed to binge-level alcohol use prenatally. This is important as a higher number of developed MRBs are associated with greater morbidity and mortality (Wright et al. 2018).

These findings may have implications for how clinicians and public health professionals explain the potential long-term risks of PAE to parents who are pregnant or planning to become pregnant in the prevention or reduction of PAE and in how clinicians may screen for hazardous alcohol use in those with PAE later in life. It is crucial, however, that any opportunities healthcare professionals’ take to discuss alcohol intake, such as in pregnant mothers, are handled with utmost empathy and a non-judgmental manner (National Institute for Health and Care Excellence 2022).

The findings of an association between PAE from binge drinking and engagement in risky sexual behavior in adolescence (underage sexual intercourse), coupled with the findings of frequent PAE being associated with hazardous alcohol use in adolescence, warrant further research to explore the potential intergenerational impacts of PAE. To investigate this, research would need to look at how the prevalence of PAE changes in subsequent generations of those with PAE. Such research may have implications for targeted health education and prevention strategies for hazardous alcohol use coupled with sexually risky behaviors in adolescents with PAE.

## Supplementary Material

**Supporting Information**

Additional supporting information can be found online in the Supporting Information section.

Supplementary Tables S1-S9; Supplementary Figures A-P

## Figures and Tables

**Table 1:** Prenatal alcohol exposure and comparator group definitions.

Type of exposure compared	Comparator/exposure(s)	Definition	Source	Questions used
Frequency of PAE	No PAE (comparator)	Adolescents with no PAE during first 3 months of pregnancy AND when baby first moved	Maternal questionnaire at 18 weeks gestation	How often have you drink alcoholic drinks in the first 3 months of this pregnancy? How often have you drink alcoholic drinks when the baby first moved? Answers: Never less than one glass* per week At least one glass* per week One to two glasses every day At least three to nine glasses* every day At least 10 glasses* every day *By glass we mean a pub measure of spirits, half a pint of lager or cider, a glass of wine etc.
	Infrequent PAE	Adolescents with infrequent PAE (< 1 glass of wine per week or equivalent) EITHER during first 3 months of pregnancy OR when baby first moved	Maternal questionnaire at 18 weeks gestation	
	Frequent PAE	Adolescents with frequent PAE (≥ 1 glass of wine per week or equivalent) EITHER during first 3 months of pregnancy OR when baby first moved	Maternal questionnaire at 18 weeks gestation	
Presence of prenatal binge-drinking exposure	No prenatal binge-drinking exposure (comparator)	Adolescents with no prenatal binge-drinking exposure reported in the last month of pregnancy prior to questionnaire completion	Maternal questionnaire at 18 weeks gestation	1. How many days in the past month have you drunk the equivalent of two pints of beer, four glasses of wine or four pub measures of spirit? Answers: None 1–2 days 3–4 days 5–10 days More than 10 days Everyday
	Prenatal binge-drinking exposure	Adolescents with prenatal binge-drinking exposure reported during the last month of pregnancy prior to questionnaire completion (2 pints of beer or more or equivalent on at least one occasion)	Maternal questionnaire at 18 weeks gestation	

**Table 2:** ALSPAC definitions of multiple risk behaviors.

Multiple risk behavior	Definition
Hazardous alcohol use	In the past year had scored 8 or more out of 40 on the Alcohol Use Disorders Identification Test (AUDIT) indicating hazardous alcohol consumption
Smoking	Has ever smoked and is regularly smoking by currently smoking at least one cigarette per week
Cannabis use	Those who reported using cannabis “sometimes but less often than once a week” or more regularly
Drug use (excluding cannabis)	In the year since their 15th birthday, young person had either been a regular user (i.e., used five or more times) of one or more illicit drugs (excluding cannabis), including amphetamines, ecstasy, LSD, cocaine, ketamine, or inhalants including aerosols, gas, solvents, and poppers
Engagement in unsafe sex	Penetrative sex without the use of contraception on the last occasion they had sex in the past year
Underage sexual intercourse	Young person has engaged in sexual intercourse in the last year, and they were aged less than 16 years old
Antisocial behavior	Young person reported that at least once in the past year they had undertaken at least one of the following seven offenses:-carried a weapon; physically hurt someone on purpose; stolen something; sold illicit substances to another person; damaged property belonging to someone else either by using graffiti, setting fire to it or destroying or damaging it in another fashion; subjected someone to verbal or physical racial abuse; or been rude/rowdy in a public place

**Table 3:** Multiple imputation sample characteristics table for the component-based MRB analysis of individuals with at least one complete MRB variable (*N* = 6752).

Maternal, child or partner characteristics	Frequency of prenatal alcohol exposure (PAE)						Prenatal binge-drinking exposure groups			
	No PAE (*N* = 2268)	Infrequent PAE (*N* = 3552)	Frequent PAE (*N* = 932)	Total (*N* = 6752)	*p* ^a^		No binge drinking (*N* = 5769)	Binge drinking (*N* = 983)	Total (*N* = 6752)	*p* ^a^
**Maternal age at delivery (years)**					< 0.001					0.20
≤ 19 years	59 (2.6%)	51 (1.4%)	9 (0.9%)	119 (1.8%)			99 (1.7%)	20 (2.0%)	119 (1.8%)	
> 19 years and ≤ 34 years	2004 (88.4%)	3045 (85.7%)	736 (78.9%)	5785 (85.7%)			4960 (86.0%)	824 (83.8%)	5785 (85.7%)	
≥ 35 years	204 (9.0%)	457 (12.9%)	188 (20.2%)	849 (12.6%)			709 (12.3%)	139 (14.1%)	849 (12.6%)	
**Any smoking during pregnancy**					< 0.001					< 0.001
No	1925 (84.9%)	2943 (82.8%)	711 (76.3%)	5579 (82.6%)			4917 (85.2%)	662 (67.3%)	5579 (82.6%)	
Yes	343 (15.1%)	609 (17.2%)	221 (23.7%)	1173 (17.4%)			852 (14.8%)	321 (32.7%)	1173 (17.4%)	
**Maternal cannabis use during pregnancy**					< 0.001					< 0.001
No	2246 (99.0%)	3476 (97.8%)	881 (94.5%)	6603 (97.8%)			5665 (98.2%)	938 (95.4%)	6603 (97.8%)	
Yes	22 (1.0%)	76 (2.2%)	51 (5.5%)	149 (2.2%)			104 (1.8%)	46 (4.6%)	149 (2.2%)	
**Maternal drug use (excluding cannabis) during pregnancy**					0.07					0.007
No	2260 (99.7%)	3543 (99.7%)	925 (99.2%)	6729 (99.7%)			5754 (99.7%)	975 (99.1%)	6729 (99.7%)	
Yes	7 (0.3%)	9 (0.3%)	7 (0.8%)	23 (0.3%)			15 (0.3%)	8 (0.9%)	23 (0.3%)	
**Parity**					< 0.001					< 0.001
Nulliparous	1182 (52.1%)	1634 (46.0%)	439 (47.1%)	3255 (48.2%)			2813 (48.8%)	442 (45.0%)	3255 (48.2%)	
Primiparous	757 (33.4%)	1304 (36.7%)	306 (32.9%)	2367 (35.1%)			2051 (35.6%)	315 (32.1%)	2367 (35.1%)	
Multiparous	329 (14.5%)	614 (17.3%)	186 (20.0%)	1129 (16.7%)			904 (15.7%)	226 (23.0%)	1129 (16.7%)	
**Maternal social class**					< 0.001					< 0.001
I to III (Non-manual)	1812 (79.9%)	2966 (83.5%)	807 (86.5%)	5585 (82.7%)			4817 (83.5%)	768 (78.1%)	5585 (82.7%)	
III (Manual) to V	455 (20.1%)	586 (16.5%)	126 (13.5%)	1167 (17.3%)			952 (16.5%)	216 (21.9%)	1167 (17.3%)	
**Mother’s highest educational qualification**					< 0.001					< 0.001
A-level or above	849 (37.4%)	1679 (47.3%)	498 (53.4%)	3026 (44.8%)			2668 (46.2%)	359 (36.5%)	3026 (44.8%)	
Lower than A-level	1419 (62.6%)	1873 (52.7%)	434 (46.6%)	3726 (55.2%)			3101 (53.8%)	625 (63.5%)	3726 (55.2%)	
**Home ownership status**					0.01						< 0.001
Own home	1851 (81.6%)	3004 (84.6%)	783 (84.0%)	5688 (83.5%)				4880 (84.6%)	758 (77.1%)	5688 (83.5%)	
No owned home or mortgage	417 (18.4%)	548 (15.4%)	149 (16.0%)	1114 (16.5%)				888 (15.4%)	226 (22.9%)	1114 (16.5%)	
**Marital status**					< 0.001						< 0.001
Married	1865 (82.3%)	2928 (82.4%)	706 (75.7%)	5499 (81.4%)				4803 (83.3%)	697 (70.8%)	5499 (81.4%)	
Not married	402 (17.7%)	624 (17.6%)	227 (24.3%)	1253 (18.6%)				966 (16.7%)	287 (29.2%)	1253 (18.6%)	
**Sex of child**					0.83						0.74
Female	1250 (55.1%)	1958 (55.1%)	504 (54.1%)	3713 (55.0%)				3167 (54.9%)	546 (55.5%)	3713 (55.0%)	
Male	1017 (44.9%)	1594 (44.9%)	428 (45.9%)	3039 (45.0%)				2602 (45.1%)	437 (44.5%)	3039 (45.0%)	
**Child birthweight**					0.10						0.27
≥2500 g	2138 (94.3%)	3393 (95.5%)	882 (94.6%)	6414 (95.0%)				5487 (95.1%)	927 (94.2%)	6414 (95.0%)	
< 2500 g	129 (5.7%)	159 (4.5%)	50 (5.4%)	338 (5.0%)				282 (4.9%)	57 (5.8%)	338 (5.0%)	
**Length of pregnancy**					0.16						0.29
**(gestational length)**											
Extreme pre-term	*	*	*	*				*	*	*	
Pre-term	*	*	*	*				*	*	*	
Term	2130 (93.9%)	3374 (95.0%)	881 (94.5%)	6385 (94.6%)				5460 (94.7%)	925 (94.0%)	6385 (94.6%)	
**Partner’s alcohol consumption**					< 0.001						< 0.001
**during pregnancy**											
Never	181 (8.0%)	71 (2.0%)	7 (0.8%)	259 (3.8%)				248 (4.8%)	12 (1.2%)	259 (3.8%)	
Infrequent	722 (31.9%)	766 (21.6%)	58 (6.2%)	1546 (22.9%)				1409 (24.4%)	137 (13.9%)	1546 (22.9%)	
Frequent	1364 (60.2%)	2715 (76.4%)	867 (93.0%)	4946 (73.3%)				4111 (71.3%)	835 (84.9%)	4946 (73.3%)	
**MRB—Hazardous alcohol use**					< 0.001						< 0.001
No	1538 (67.8%)	2195 (61.8%)	499 (53.6%)	4232 (62.7%)				3687 (63.9%)	545 (55.4%)	4232 (62.7%)	
Yes	730 (32.2%)	1357 (38.2%)	433 (46.4%)	2520 (37.3%)				2082 (36.1%)	438 (44.6%)	2520 (37.3%)	
**MRB—Drug use (excluding cannabis)**					0.02					< 0.001
No	2136 (94.2%)	3330 (93.7%)	853 (91.5%)	6319 (93.6%)			5425 (94.0%)	894 (90.9%)	6319 (93.6%)	
Yes	132 (5.8%)	222 (6.3%)	79 (8.5%)	433 (6.4%)			344 (6.0%)	89 (9.1%)	433 (6.4%)	
**MRB—Cannabis use**					< 0.001					0.002
No	2077 (91.6%)	3159 (88.9%)	809 (86.8%)	6045 (89.5%)			5192 (90.0%)	853 (86.7%)	6045 (89.5%)	
Yes	190 (8.4%)	393 (11.1%)	123 (13.2%)	707 (10.5%)			577 (10.0%)	130 (13.3%)	707 (10.5%)	
**MRB—Regular smoking**					0.10					< 0.001
No	1965 (86.6%)	3081 (86.8%)	784 (84.1%)	5830 (86.3%)			5040 (87.4%)	791 (80.4%)	5830 (86.3%)	
Yes	303 (13.4%)	470 (13.2%)	148 (15.9%)	922 (13.7%)			729 (12.6%)	193 (19.6%)	922 (13.7%)	
**MRB—Antisocial behavior**					0.06					< 0.001
No	1263 (55.7%)	1874 (52.8%)	487 (52.2%)	3624 (53.7%)			3153 (54.7%)	472 (48.0%)	3624 (53.7%)	
Yes	1004 (44.3%)	1678 (47.2%)	445 (47.8%)	3128 (46.3%)			2616 (45.3%)	512 (52.0%)	3128 (46.3%)	
**MRB—Unsafe sex**					0.56					0.07
No	2222 (98.0%)	3488 (98.2%)	919 (98.6%)	6629 (98.2%)			5671 (98.3%)	958 (97.4%)	6629 (98.2%)	
Yes	45 (2.0%)	64 (1.8%)	13 (1.4%)	122 (1.8%)			98 (1.7%)	25 (2.6%)	122 (1.8%)	
**MRB—Underage sex**					0.88					< 0.001
No	1880 (82.9%)	2932 (82.6%)	766 (82.2%)	5578 (82.6%)			4828 (83.7%)	751 (76.3%)	5578 (82.6%)	
Yes	388 (17.1%)	620 (17.4%)	166 (17.8%)	1174 (17.4%)			941 (16.3%)	233 (23.7%)	1174 (17.4%)	

*Note:* The * sign indicates suppressed counts of < 5 people, or counts where a suppressed number can be calculated, for example, from the percentage of another count. Participant counts may not sum to the row total *N* nor the column header total *N*, as values are based on pooled multiple imputation estimates rounded to the nearest integer. Column header totals are also rounded imputed values with the exception of the sample total *N* = 6752. Percentages by covariate or MRB group may not total 100% due to percentage rounding to 1 decimal place. Abbreviations: MRB, multiple risk behavior; PAE, prenatal alcohol exposure.

a *p* values calculated using chi-squared analyses for categorical variables.

**Table 4:** Descriptive statistics of passively imputed adolescent MRB scores by prenatal alcohol exposure groups for those with at least one complete MRB variable (*N* = 6752)

Child characteristics	Frequency of prenatal alcohol exposure (PAE)						Prenatal binge-drinking exposure groups			
	No PAE (*N* = 2269)	Infrequent PAE (*N* = 3553)	Frequent PAE (*N* = 930)	Total (*N* = 6752)	*p* ^a^		No binge drinking (*N* = 5770)	Binge drinking (*N* = 982)	Total (*N* = 6752)	*p* ^a^
**MRB score**					< 0.001					< 0.001
0	1079 (47.6%)	1496 (42.1%)	358 (38.5%)	2933 (43.4%)			2567 (44.5%)	367 (37.3%)	2933 (43.4%)	
1	414 (18.2%)	717 (20.2%)	169 (18.2%)	1300 (19.3%)			1131 (19.6%)	170 (17.3%)	1300 (19.3%)	
2	367 (16.2%)	629 (17.7%)	188 (20.2%)	1184 (17.5%)			994 (17.2%)	190 (19.4%)	1184 (17.5%)	
3	169 (7.4%)	311 (8.8%)	93 (10.0%)	573 (8.5%)			480 (8.3%)	93 (9.5%)	573 (8.5%)	
4	82 (3.6%)	171 (4.8%)	48 (5.2%)	291 (4.3%)			246 (4.3%)	56 (5.7%)	291 (4.3%)	
5	70 (3.1%)	117 (3.3%)	38 (4.1%)	225 (3.3%)			174 (3.0%)	51 (5.2%)	225 (3.3%)	
6	68 (3.0%)	89 (2.5%)	31 (3.3%)	188 (2.8%)			140 (2.4%)	47 (4.8%)	188 (2.8%)	
7	20 (0.9%)	23 (0.6%)	5 (0.6%)	48 (0.7%)			38 (0.7%)	10 (1.0%)	48 (0.7%)	

*Note:* Participant totals may slightly differ from Table 3, as they are estimates derived from separate imputation models leading to inherent variability. Participant counts of MRB scores may not sum to the row total *N* nor the column header total *N*, as values are based on pooled multiple imputation estimates rounded to the nearest integer. Column header totals also rounded imputed values with the exception of the sample total *N* = 6752. Percentages by covariate or MRB group may not total 100% due to percentage rounding to 1 decimal place. Abbreviations: MRB, multiple risk behaviors; PAE, prenatal alcohol exposure.

a *p* values calculated using chi-squared analysis for categorical (ordinal) variables.

**Table 5:** Multiple imputation results (*N* = 6752) comparing adjusted MRB score between PAE exposure and comparator groups.

PAE exposure group	Adjusted MRB score difference (coefficient) (95% CI)	*p*	*q* value (if *p* < 0.05)
Infrequent PAE (compared with no alcohol)	(+)0.05 (–0.04 to +0.14)	0.53	—
Frequent PAE (compared with no alcohol)	(+)0.13 (–0.01 to +0.27)	0.07	—
Binge drinking (compared with no binge drinking)	(+)0.21 (+0.08 to +0.33)	0.001	0.02

**Table 6:** Multiple imputed (*N* = 6752) aORs of MRBs in PAE exposure groups relative to comparator groups.

Multiple risk behavior at the age of 16	Infrequent PAE (compared with no PAE)				Frequent PAE (compared with no PAE)				Binge drinking (compared with no binge drinking)		
	Adjusted odds ratio (95% CI)	*p*	*q* value (if *p* < 0.05)		Adjusted odds ratio (95% CI)	*p*	*q* value (if *p* < 0.05)		Adjusted odds ratio (95% CI)	*^p^*	*q* value (if *p* < 0.05)
Hazardous alcohol use	1.17 (1.01–1.35)	0.03	0.17		1.45 (1.19–1.76)	< 0.001	0.005		1.17 (0.97–1.42)	0.10	—
Regular smoking	0.97 (0.79–1.18)	0.74	—		1.10 (0.84–1.45)	0.48	—		1.29 (1.03–1.61)	0.03	0.16
Regular cannabis use	1.22 (0.97–1.53)	0.10	—		1.22 (0.89–1.67)	0.20	—		1.11 (0.84–1.45)	0.45	—
Drug use	1.01 (0.75–1.37)	0.94	—		1.19 (0.79–1.77)	0.40	—		1.28 (0.92–1.77)	0.14	—
Antisocial behavior	1.12 (0.99–1.27)	0.08	—		1.07 (0.89–1.28)	0.49	—		1.15 (0.98–1.36)	0.09	—
Unsafe sexual intercourse	0.96 (0.56–1.64)	0.87	—		0.77 (0.36–1.68)	0.52	—		1.32 (0.73–2.40)	0.35	—
Underage sexual intercourse	1.00 (0.84–1.20)	0.96	—		0.98 (0.75–1.28)	0.88	—		1.34 (1.09–1.64)	0.005	0.04

*Note:* Adjustment model covariates were maternal age, parity, birthweight, maternal social class, marital status, home ownership status, highest maternal educational qualification, maternal smoking, maternal cannabis use, maternal drug use (excluding cannabis), and paternal alcohol use during pregnancy.

## Data Availability

The data that support the findings of this study are available from ALSPAC. Restrictions apply to the availability of these data, which were used under license for this study. Data are available from https://www.bristol.ac.uk/alspac/researchers/access/ with the permission of ALSPAC.
